# Immunoexpression of Trefoil Factor 1 in Non-Neoplastic and Neoplastic Canine Gastric Tissues

**DOI:** 10.3390/ani11102855

**Published:** 2021-09-29

**Authors:** Ana R. Flores, Marisa Castro, Alexandra Rêma, João R. Mesquita, Marian Taulescu, Fátima Gärtner, Fernanda Seixas, Irina Amorim

**Affiliations:** 1 Department of Pathology and Molecular Immunology of the Institute of Biomedical Sciences Abel Salazar (ICBAS), University of Porto, 4050-313 Porto, Portugal; anaruteflores@gmail.com (A.R.F.); mmcastro@icbas.up.pt (M.C.); alexandra.rema@gmail.com (A.R.); fgartner@ipatimup.pt (F.G.); iamorim@ipatimup.pt (I.A.); 2 Institute of Pathology and Molecular Immunology of the University of Porto (IPATIMUP), 4200-465 Porto, Portugal; 3 Animal and Veterinary Research Center (CECAV), Associate Laboratory AL4AnimalS, University of Trás-os-Montes e Alto Douro (UTAD), 5001-801 Vila Real, Portugal; fseixas@utad.pt; 4 Epidemiology Research Unit (EPIUnit), Instituto de Saúde Pública da Universidade do Porto (ISPUP), 4050-600 Porto, Portugal; jmesquita@outlook.com; 5 Department of Pathology, Faculty of Veterinary Medicine, University of Agricultural Sciences and Veterinary Medicine, 400372 Cluj-Napoca, Romania; 6 Synevovet Laboratory, 81 Pache Protopopescu, 021408 Bucharest, Romania; 7 i3S-Instituto de Investigação e Inovação em Saúde, Universidade do Porto, 4200-135 Porto, Portugal

**Keywords:** dog, gastric carcinoma, gastric polyps, trefoil factor 1, stomach

## Abstract

**Simple Summary:**

Gastric carcinoma (GC) is the second leading cause of death in humans and the most frequent malignancy in the stomach of dogs. As in humans, the prognosis of canine gastric cancer is generally poor owing to the advanced stage at the time of diagnosis, resulting in limited treatment options. In dogs, the molecular mechanisms involved in the growth and progression of gastric cancer remain largely unknown. Trefoil factor 1 (TFF1) protein is a mucin-associated secretory molecule that plays an important role in the maintenance and protection of epithelial surface integrity. Some human studies showed that TFF1 can protect mucosa against damage and suppress carcinogenesis, while other studies showed that TFF1 can restrict cell adhesion, promote tumor cell invasion, and block necrosis of tumor cells. In human gastric cancer, TFF1 has been found to decrease, and it has been proposed that it might act as a tumor suppressor factor. The present study was carried out to investigate whether there is a relationship between TFF1 and canine gastric carcinogenesis. We found an association between reduced expression of TFF1 and the development and progression of gastric cancer in dogs. The pathological and behavioral similarities between spontaneous canine GC and human counterparts make it logical to assume that dogs may be a useful model for human gastric cancer.

**Abstract:**

TFF1 expression is markedly reduced in human GCs, suggesting that TFF1 is a tumor suppressor for human gastric cancer. The present study evaluated the expression and distribution pattern of TFF1 in paraffin-embedded canine gastric tissue samples, including normal mucosa (n = 3), polyps (n = 8), carcinomas (n = 31) and their adjacent non-neoplastic mucosa (n = 30), neoplastic emboli (n = 14), and metastatic lesions (n = 9), by immunohistochemistry (IHC). All normal gastric tissues expressed TFF1 in the superficial foveolar epithelium and mucopeptic cells of the neck region. Most gastric polyps (GPs) displayed immunoreactivity for TFF1 in >75% of the epithelial component. In GCs, the expression of TFF1 was found reduced in 74.2% of the cases. The level of TFF1 expression had a decreased tendency from normal gastric mucosa to GPs and GCs (*p* < 0.05). No significant differences in the expression of TFF1 were found in GCs, according to age, sex, histological type based on World Health Organization (WHO) and Lauren classification, tumor location, depth of tumor invasion, presence of neoplastic emboli or metastatic lesions. The median survival time of GC patients with preserved and reduced TFF1 immunoexpression were 30 and 12 days, respectively. Kaplan–Meier analysis revealed no significant survival differences between the two groups (*p >* 0.05). These findings suggest that TFF1 protein may play a role in canine gastric carcinogenesis, and further studies are necessary to define its usefulness as a prognostic indicator in canine gastric carcinoma.

## 1. Introduction

Gastric carcinoma is the most frequent malignant neoplasm of the stomach in dogs, comprising 50–90% of all canine gastric malignancies, and usually results in death. Although, in recent years this canine disease appears to have been diagnosed more frequently, prognosis is generally poor owing to the advanced stage at the time of diagnosis, resulting in limited treatment options [1]. Treatment involves surgical resection that is often complicated by diffuse infiltration, metastasis, and a frequently debilitated patient [1,2,3]. 

The WHO for domestic animals subdivided GCs into papillary, tubular, mucinous, signet ring cell, and undifferentiated types [4]; however, previous studies demonstrated that some canine gastric neoplastic lesions fit specific histological types only described in the human WHO classification, such as poorly cohesive and mixed carcinomas [1,5]. Despite its usefulness in the recognition of the morphological patterns, WHO classification schemes offer little prognostic significance [4,6]. In turn, the human Lauren classification may more accurately predict prognosis based on histopathological features and has been successfully adapted to the dog [7,8,9,10].

In humans, studies emphasize that GPs may be intermediate steps in the process of malignant transformation leading to specific histological types of GC [11,12]. According to the current veterinary literature, there are no validated pre-neoplastic lesions of GC, although atrophic gastritis, a type of giant hypertrophic gastropathy or Ménétrier’s disease, and intestinal metaplasia (IM) have been recognized in dogs and associated with possible predisposition to gastric cancer development [2,13,14,15]. As in humans, GPs are incidental findings in dogs; however, previous studies conducted by our group suggest that canine GPs are proliferative and hyperplastic lesions with lack of features suggestive of neoplastic transformation [10,16]. 

Trefoil factor peptides (TFFs) are a family of mucin-associated secretory molecules that have an important role in the maintenance and protection of epithelial surface integrity [17]. They are secreted in response to injuries, acting as mitogens to facilitate cell migration into the lesion, forming a protective barrier, and thus being crucial for epithelial restitution, particularly of mucosal surfaces. In addition, TFFs have been described as potent inhibitors of apoptosis and anoikis (cell death induced by anchorage independence) [18]. 

In mammals, the TFF family consists of three members: TFF1 (previously pS2), TFF2 (formerly spasmolytic polypeptide or SP), and TFF3 (or intestinal trefoil factor). TFF peptides possess a sequence of about 40 amino acids termed TFF domain (formerly: P-domain, trefoil domain) that contains six conserved cysteine residues with three intramolecular disulfide bonds (Cys^I-V^, Cys^II-IV^, and Cys^III-VI^). Both, TFF1 and TFF3 have one trefoil domain, while TFF2 has two trefoil domains [17]. 

Trefoil factor 1, a 6.7kDa protein of 60 amino acids, was originally isolated from estrogen-induced human breast cancer cell line MCF-7 [19,20] and is normally expressed in the superficial and foveolar epithelium of the gastric mucosa and in the upper ducts of Brunner’s glands in the duodenum in both rodents and humans [21,22]. Additionally, TFF1 was also detected in the gastric juice [17].

In recent years, clinical and experimental studies have shown an active function of the TFF1 in the oncogenic transformation, growth, and metastatic extension of common human solid tumors, including breast, pancreas, colon, and stomach cancer [23]. Animal experiments showed that TFF1 knockout mice develop marked hyperplasia and dysplasia of gastric cells, antral/pylorus-specific adenoma and, in 30% of cases, multifocal intraepithelial or intramucosal carcinoma [24]. In human gastric tissues, the expression of TFF1 has been found decreased in IM and adenomas compared with adjacent normal mucosa, and completely lost in about 40% to 60% of the carcinomas [12,25,26,27]. Thus, it has been proposed that TFF1 functions as a gastric tumor suppressor gene [28]. 

The genes encoding TFFs were previously characterized in dogs and cats [29]. Schmitz et al. [30] assessed TFF gene expression in the gastrointestinal tract from dogs with inflammatory bowel disease (IBD) by PCR and demonstrated that TFF1 expression was significantly upregulated in duodenum of dogs with inflammatory bowel disease (IBD). Very little is known regarding TFF1 in canine gastric tissues.

The aims of the present study were: (1) to evaluate the expression and distribution pattern of TFF1 in normal gastric mucosa, GPs, and malignant gastric tumors with neoplastic emboli and corresponding metastasis in dogs; (2) to determine whether there was an association between the expression of this protein and clinicopathological features of the tumors and patient’s survival time. 

## 2. Materials and Methods

### 2.1. Ethics Statement

All the examined samples were collected for diagnostic purposes as part of routine standard care based on the best clinical judgement of their attending practitioners, and the investigators had no influence on the execution of any clinical procedures. Owners gave informed consent to use clinical data and the excised tissues for teaching and research purposes. The study was approved by Animal Welfare Organization (ORBEA) of the ICBAS-UP (Porto, Portugal), authorization Nº 201/2017.

### 2.2. Sample Collection

Thirty-nine canine gastric lesions, obtained during endoscopic procedures, surgery, or necropsy examination between 2004 and 2021, were selected from the archives of the Laboratory of Veterinary Pathology, ICBAS-UP (Porto, Portugal). These included 8 GPs and 31 GCs. Regarding GCs, full-thickness biopsies were performed in 22 cases (71%); partial biopsies including mucosa, submucosa, and tunica muscularis were achieved in 6 cases (19.4%); in the remaining 3 cases, partial biopsies were performed, which included the mucosal and submucosal layers (9.7%). Samples of normal canine body and antral gastric mucosa were collected during necropsy examination of three animals that died of causes not related with gastrointestinal diseases. 

Epidemiological and clinical data of the dogs diagnosed with gastric disease was collected from the histopathological request forms (Table 1). When available, medical records were also reviewed to obtain the outcome of gastric cancer patients.

### 2.3. Histological Evaluation

Tissues were fixed in 10% buffered formalin and paraffin embedded. Serial consecutive 2 μm-thick sections were made; one being stained with Hematoxylin and Eosin (H&E, Merck, Darmstadt, Germany) for histological diagnosis and the other for the immunohistochemical study.

All sections, including normal gastric samples, GPs, and GCs were independently examined by three veterinary pathologists. When there was a disagreement, a consensual diagnosis was achieved through simultaneous observation using a multi-head microscope. If this approach was not possible for some reason, and since the slides of all cases were scanned, pathologists reached consensus through their joint, but at a distance, virtual reassessment.

Normal gastric tissues were considered as such according to the previously proposed criteria [33] and were negative for the presence of *Helicobacter* spp. (confirmed by modified Giemsa stain and IHC using anti-*H. pylori* polyclonal antibody (RBK012; Zytomed, Berlin, Germany, diluted 1:200)).

Gastric polyps were classified according to WHO classification for domestic animals’ diagnostic criteria [4]. 

GC cases were reclassified according to the diagnostic criteria of the human WHO (2010) [31] since WHO classification of digestive system tumors for domestic animals does not include all the histological subtypes recognized in this study. Canine GCs were classified as tubular when they contained prominent neoplastic tubules; as papillary when neoplastic cells formed papillary structures; mucinous when they contained >50% of extracellular mucin and signet ring cell and when the great majority of the tumor was composed of malignant cells containing intracytoplasmic mucin vacuoles and eccentric nuclei. Additionally, two other neoplastic malignant entities were considered: poorly cohesive carcinomas when lesions were mainly composed of poorly cohesive cells, morphologically resembling histiocytes, lymphocytes and plasma cells; and mixed carcinomas when they contained a mixture of well differentiated and signet ring/poorly cohesive histological components [1,31]. Tumors were further classified according to Lauren classification into the following categories: intestinal type when they contained rudimentary glands that superficially resembled intestinal glands; diffuse type when they contained cells that failed to form distinct structures; indeterminate type when they contained equal proportions of intestinal and diffuse characteristics [32]. For statistical purposes, tumors were divided into two main histological subgroups: differentiated type consisting of papillary and tubular carcinomas, and poorly/undifferentiated type consisting of mucinous, signet ring cell, poorly cohesive, and mixed carcinomas. The anatomic location was determined by combining the clinical information and imaging exams provided by the assistant veterinarian with the analysis of the gastric tissue fragments sent for histopathological analysis, which in most cases allowed the confirmation of the gastric region affected. The depth of tumor invasion of the gastric wall was recorded in every case as the deepest layer invaded: mucosa, submucosa, tunica muscularis, and serosa. However, for statistical analysis only cases that included all layers of the gastric wall (full-thickness biopsies) were considered.

### 2.4. Immunohistochemistry

For IHC, sections were deparaffinized, hydrated, and antigen retrieval was performed in a water bath in 10% citrate buffer, pH 6, for 20 min. The NovolinkTM Max-Polymer detection system (Novocastra, Newcastle, UK) was used for visualization, according to the manufacturer’s instructions. Slides were incubated with anti-estrogen inducible protein pS2 rabbit monoclonal antibody (clone EPR3972, ab92377, Abcam, Cambridge, UK), diluted 1:2000, overnight at 4 °C in a humid chamber. Sections were rinsed with triphosphate buffered saline (TBS, Cell marque, Merck, Darmstadt, Germany) in each step of the procedure. Color was developed with 3.3- diamino-benzidine (DAB; Sigma, St. Louis, MO, USA) and sections were then counterstained with hematoxylin, dehydrated, and mounted. Positive control tissues were represented by sections of human gastric mucosa with IM, obtained from the pathology department archive of Hospital Santo Antonio (Porto, Portugal), known to express TFF1. Negative controls were performed by replacing the primary antibody by an antibody of the same immunoglobulin isotype at the same concentration as the former. 

All immunostained slides were independently evaluated by three observers in blind testing—without the knowledge of the specific diagnosis or prognosis for each individual case. When there was a disagreement, a consensual diagnosis was achieved through simultaneous observation using a multi-head microscope. In normal canine gastric mucosa, the TFF1 immunolabeling was evaluated at the superficial foveolar epithelium and glandular structures of both body and antral gastric regions. In GPs, TFF1 immunoexpression was evaluated in all epithelial components. In GCs, the TFF1 immunoreactivity was evaluated in both neoplastic epithelial cells and in non-neoplastic gastric mucosa adjacent to carcinomas.

TFF1 immunoreactivity was scored semi-quantitatively according to the presence of immunoreactive cells as: −, none or rare positive cells (<5%); +, 5–25%; ++, 25–75%; +++, >75% [34]. The subcellular location of the immunostaining was classified as cytoplasmic (diffuse or apical) or membranous. For further statistical analysis, TFF1 immunoexpression was grouped into preserved (+++) or reduced (−, +, ++), in comparison with the immunoreactivity observed in normal canine gastric mucosa.

### 2.5. Protein Extraction and Western Blotting

To validate the specificity and cross-reactivity of this specific antibody with canine tissues, a Western blot analysis was performed in a sample of normal canine gastric mucosa. Briefly, the proteins were extracted from the formalin-fixed and paraffin-embedded tissues (FFPE) using the Qproteome FFPE tissue kit (Qiagen, Germantown, MD, USA). The amount of protein in each extract was estimated with Pierce^®^ BCA Protein Assay Kit (Thermoscientific, Rockford, IL, USA) and 20 μg of protein were separated in 8% acrylamide/bis acrylamide (Sigma, St. Louis, MO, USA) SDS-PAGE gel. The gel was transferred onto a nitrocellulose membrane, blocked with 5% skim milk for 1 h at room temperature, and incubated with anti-estrogen inducible protein pS2 rabbit monoclonal antibody (clone EPR3972, ab92377, Abcam, Cambridge, UK), diluted 1:5000 overnight at 4 °C. The membrane was then washed with TBS for 30 min and incubated with horseradish peroxidase (HRP)-conjugated anti-mouse secondary antibody (Thermoscientific, Rockford, IL, USA), diluted 1:10,000 in 1% skim milk for 1 h at room temperature. After washing, the bound antibody was revealed by chemiluminescence using the ECL prime Kit (Bio-Rad, Hercules, CA, USA).

### 2.6. Statistical Analysis

The chi-square test and chi-square test for trend were used to assess the association between TFF1 expression and histological type and the various clinicopathological features of the tumors. The survival time was defined as the interval in days between the date of diagnosis and the date of animal death by natural death and/or euthanasia. Whenever euthanasia was performed during surgery or for reasons related to the deterioration of the animal´s health conditions due to the progression of the neoplastic disease, the cause of death was considered to be related to the tumor. Survival time was censored for dogs that were lost to follow up. Kaplan–Meier survival analyses with log-rank test was performed to compare the differences between the median survival time of the two groups preserved and reduced TFF1 immunoexpression. Differences were considered statistically significant at values of *p* < 0.05. Analyses were performed using GraphPad Prism 5 (GraphPad Software Inc., La Jolla, CA, USA).

## 3. Results

### 3.1. Case Details

The available epidemiological data (breed, age, sex) and characteristics of the lesions are summarized in Table 1.

The mean age of dogs diagnosed with GPs was 12.1 years ± 2.6 (range 9–16 years), and the male: female ratio was 5:3. Of the eight GPs, seven were located in the antral region and one at the body region. Five out of eight GPs were classified as hyperplastic polyps and three as inflammatory polyps. All GPs revealed *Helicobacter* organisms, preferentially located in the hyperplastic foveolae and the lumen of gastric glands.

**Figure 1:**
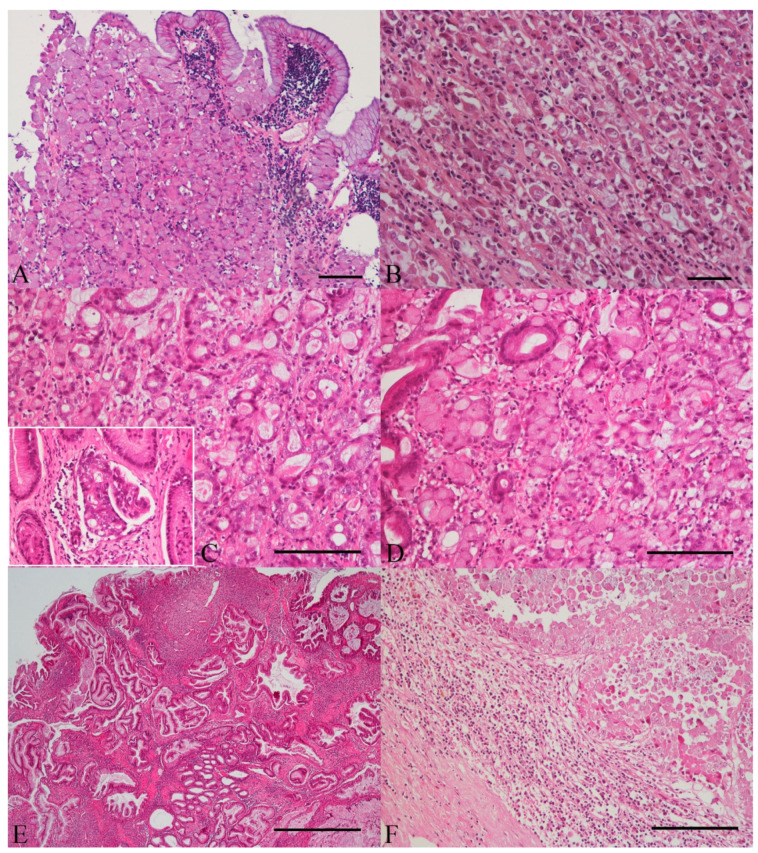
Histopathological features of gastric carcinomas and lymph node metastasis. (**A**) Signet ring cell carcinoma (WHO) and diffuse type carcinoma (Lauren) constituted by signet ring tumor cells replacing gastric mucosa (bar = 100 µm). (**B**) Poorly cohesive carcinoma (WHO) and diffuse type carcinoma (Lauren) composed of poorly cohesive tumor cells (bar = 100µm). (**C**,**D**) Mixed carcinoma (WHO) and indeterminate type carcinoma (Lauren) composed of a mixture of well-differentiated (tubules of various sizes, **C**) and signet ring/poorly cohesive histological components (**D**) (bar = 100 µm). Inset in **C** shows neoplastic emboli (200×). (**E**) Tubular carcinoma (WHO) and intestinal type carcinoma (Lauren) characterized by distended, diffuse, or branching tubules of various sizes, sometimes with intraluminal mucus (bar = 500 µm). (**F**) Lymph node metastasis of a poorly cohesive carcinoma (WHO) and diffuse type carcinoma (Lauren) with large clusters of neoplastic epithelial cells. Few aggregates of lymphoid cells are still discernible at the periphery (bar = 100 µm).

Gastric carcinomas included in this study were obtained from 19 males (61.3%) and 12 females (38.7%), with a mean age 10.1 years ± SD 2.6 (range 5–14 years old). There were five crossbreed (16.1%), four chow-chow (12.9%), two poodle, two siberian husky, two labrador retriever, two golden retriever, two collie, and twelve dogs of other breeds. Tumors were located in antral region in sixteen cases (51.6%), in twelve cases in the gastric body (38.7%), in two cases (6.5%) the lesion affected both regions, and in the remaining case (3.2%) it was not possible to determine the location due to the small size of the biopsy sample. There were six well-differentiated carcinomas (five tubular carcinomas and one papillary carcinoma) and twenty-five poorly/undifferentiated carcinomas (ten signet ring cell carcinomas, eight poorly cohesive carcinomas, five mixed carcinomas, and two mucinous carcinomas). According with Lauren classification, there were six intestinal type, twenty diffuse type, and 5 indeterminate type carcinomas (Figure 1A–E). In two cases, concerning one papillary carcinoma and one signet ring cell carcinoma, there were foci of IM. Regarding to depth of tumor invasion, fifteen carcinomas (48.4%) invaded the tunica muscularis, twelve (38.7%) the serosal layer, three (9.7%) were limited to mucosa, and one (3.2%) to submucosal layer of the gastric wall. There were 18 (58.1%) cases of GC with neoplastic emboli; however, only 14 neoplastic emboli were available for IHC evaluation. In the remaining four cases, TFF1 analysis in neoplastic emboli was not possible due to tissue exhaustion. Metastatic lesions were diagnosed in nine out of thirty-one cases of GCs. Single and isolated metastases were found in five animals affecting regional lymph nodes (n = 3) (Figure 1F), small intestine (n = 1), and liver (n = 1); another dog presented both lymph node and esophagus involvement (n = 1); in the other cases metastases were identified in multiple organs, such as lymph node, pancreas, and intestine (n = 1), intestine, peritoneum, and liver (n = 1), and lung, esophagus, liver, and adrenal gland (n = 1). For the immunohistochemical study nine metastatic samples (three intestinal, three regional lymph nodes, one esophagus, one pulmonary, and one adrenal gland metastasis) from eight dogs were also included. Tissue selection criteria were based on the amount of tissue and its conservation conditions.

During the study, 21 dogs had died and 10 were lost for follow up after diagnosis. In those whose follow-up data was accomplished, the mean survival time was 31.2 ± 40.3 days (0–150 days).

### 3.2. Specificity of the Monoclonal Antibody

The results of Western blot analysis are shown in Figure S1. Anti-estrogen inducible protein pS2 rabbit monoclonal antibody recognized a dominant band near 15 and 20 kDa in the normal canine gastric tissue sample, confirming the specificity of this specific antibody for this species.

### 3.3. Immunohistochemistry

#### 3.3.1. Normal Gastric Mucosa

**Figure 2:**
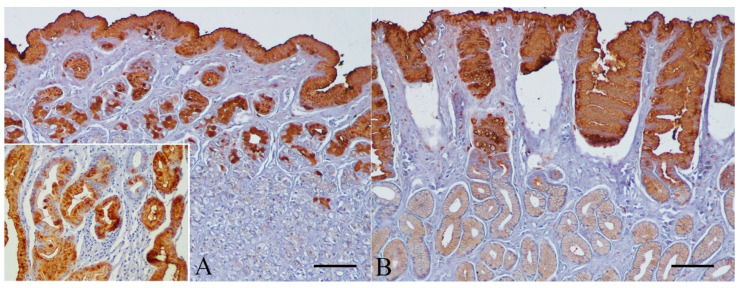
Normal canine gastric mucosa. Immunohistochemistry for TFF1 counterstained with Mayer´s hematoxylin. Strong, diffuse, and cytoplasmic TFF1 expression in superficial foveolar epithelium and mucopeptic cells of the neck region from the gastric body (**A**) and pyloric antrum (**B**). Note the absence of staining in body glands (**A**) and the decrease in labeling intensity in the antral glands (**B**) (bar = 100 µm). Inset shows diffuse cytoplasmic TFF1 immunostaining in superficial foveolar epithelium and in the foci of IM of the human gastric mucosa (positive control; 200×).

The expression of TFF1 was consistently detected in all normal canine gastric samples (100%), covering more than 75% of the superficial foveolar epithelium and mucopeptic cells of the neck region from both the gastric body (Figure 2A) and antrum (Figure 2B). The predominant staining pattern was diffuse cytoplasmic; a more intense immunostaining was also observed in the apical region of the epithelial cells along the mucosal surface and in the gastric foveolae. Glands of the body region did not show any TFF1 immunoreactivity (Figure 2A). TFF1 immunostaining was also seen in the antral glands, but the intensity of staining was much weaker than the superficial part of the mucosa (Figure 2B).

#### 3.3.2. Gastric Polyps

**Figure 3:**
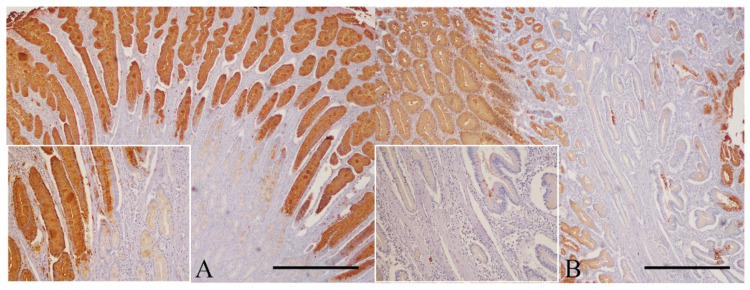
Gastric polyps. Immunohistochemistry for TFF1 counterstained with Mayer´s hematoxylin. (**A**) Hyperplastic polyp: although with different intensity, TFF1 expression is observed in all epithelial components (bar = 500 µm). Inset shows a higher magnification of TFF1 immunostaining covering superficial epithelium and antral glands (200×). (**B**) Inflammatory polyp: some glands do not display TFF1 expression, in contrast with the strong immunolabeling of superficial gastric mucosa (bar = 500 µm). Inset high shows the reduced expression of TFF1 in the antral glands (200×).

All the GPs analyzed presented TFF1 immunoexpression (100%). In hyperplastic polyps, TFF1 was detected as a diffuse cytoplasm staining in more than 75% of the cells (Figure 3A). Inflammatory polyps also presented similar subcellular location but affecting more than 75% of the cells in two cases (2/3, 66.7%) and 25–75% in 1/3 (33.3%) (Figure 3B). No relevant difference was detected between the scored encountered in these lesions and that found in normal gastric tissue.

#### 3.3.3. Non-neoplastic Gastric Mucosa Adjacent to Carcinomas

Non-neoplastic gastric mucosa adjacent to carcinomas was present in 30 out of 31 cases and in all cases, TFF1 immunoexpression was similar to that observed in normal canine gastric mucosa (100%). In four of these cases, an intense apical membrane labeling pattern was also detected in the antral glands.

#### 3.3.4. Gastric Carcinomas

**Figure 4:**
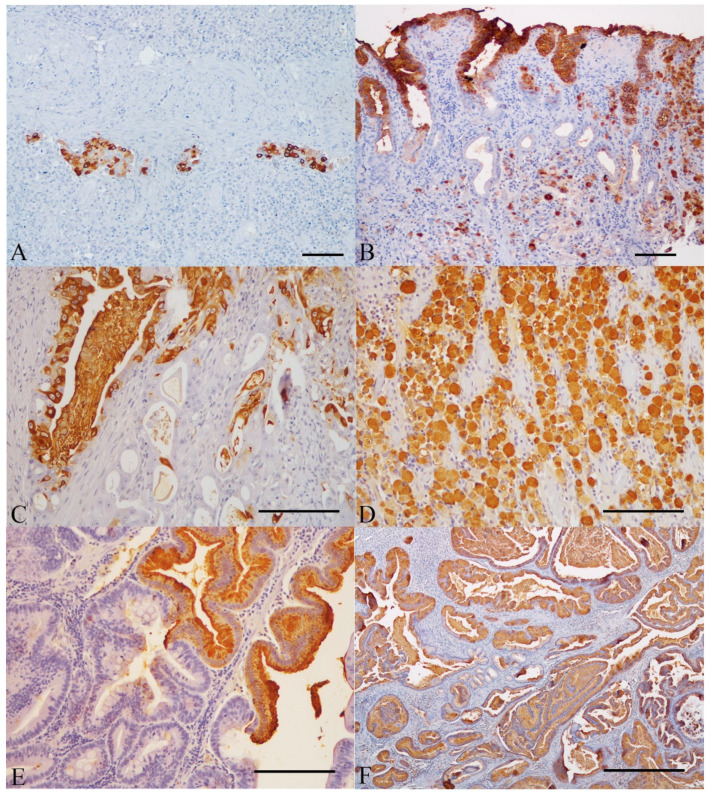
Gastric carcinomas. Immunohistochemistry for TFF1 counterstained with Mayer´s hematoxylin. (**A**) Absence of TFF1 expression (<5% of immunoreactive cells) in a poorly cohesive carcinoma (WHO) and diffuse type carcinoma (Lauren) and increased expression of TFF1 in neoplastic emboli (bar = 100 µm). (**B**) Reduced expression of TFF1 (25–75% of immunoreactive cells) in a signet ring cell carcinoma (WHO) and diffuse type carcinoma (Lauren) (bar = 100 µm). Note the intense TFF1 expression in adjacent superficial gastric mucosa. (**C**,**D**) Reduced expression of TFF1 (25–75% of immunoreactive cells) in a mixed carcinoma (WHO) and indeterminate type carcinoma (Lauren). Note reduced expression of TFF1 (25–75% of immunoreactive cells) in the intestinal (tubular) component while in diffuse component the expression was preserved (>75% of immunoreactive cells) (bar = 100 µm). (**E**) No TFF1 expression was observed in the foci of IM in a papillary carcinoma (WHO) and intestinal type carcinoma (Lauren) (bar = 100 µm). (**F**) Preserved expression of TFF1 (>75% of immunoreactive cells) in a tubular carcinoma (WHO) and intestinal type carcinoma (Lauren) (bar = 500 µm).

Twenty-three out of the 31 malignant lesions (74.2%) showed reduced TFF1 expression, characterized by absence of staining in five cases (all diffuse type carcinomas) (Figure 4A) and decreased expression in eighteen cases (eleven diffuse type, four indeterminate type, and three intestinal type carcinomas) (Figure 4B–E), compared with that observed in normal gastric mucosa. In the remaining eight cases, TFF1 expression was preserved (four diffuse type, three intestinal type, and one indeterminate type carcinomas) (Figure 4F). At the subcellular level, the pattern of TFF1 was mainly diffuse cytoplasmic, but occasionally immunostaining was also seen in apical membrane and luminal secretions of the intestinal (tubular) component. The expression of TFF1 was observed to gradually decrease from mucosa to deeper layers of the gastric wall. In the foci of IM, no TFF1 immunoexpression was recorded in one case (Figure 4E). In the other case, it was not possible to evaluate TFF1 expression in IM due to tissue exhaustion.

**Figure 5:**
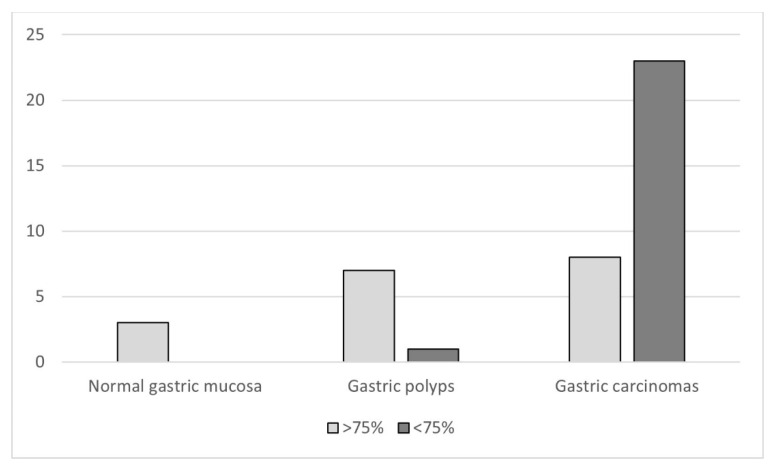
Expression of TFF1 by histological type (*p* = 0.0003).

The level of TFF1 expression showed a decreased tendency from normal gastric mucosa (100%) to GPs (87.5%) and to GCs (25.8%) and this reduction in protein expression was statistically significant (*p* = 0.0003; Figure 5). 

Table 2 summarizes the results of association analysis between clinicopathological features of the tumors and TFF1 immunoexpression. Reduced expression of TFF1 was more frequent, though not significantly, in poorly/undifferentiated carcinomas (80%) and, in indeterminate and diffuse type carcinomas (80% and 80%, respectively) than in well-differentiated or intestinal type carcinomas (50%, *p* = 0.132 and *p* = 0.229). 

Fifteen out of the 23 (65.2%) carcinomas with reduced expression of TFF1 presented neoplastic emboli, but it was not statistically significant (*p =* 0.171). Although not statistically significant, most GCs cases with the presence of metastatic disease (88.9%) displayed a reduced expression of TFF1 (*p* = 0.186). The labeling pattern of TFF1 in neoplastic emboli and metastases was further compared with that found on the primary lesion. Nine out of fourteen neoplastic emboli showed higher expression, two had similar expression, and in three cases the expression was lower than in the primary tumors. Compared to primary neoplasms, four metastatic lesions exhibited similar expression, three had lower expression and two showed higher expression.

When comparing survival times between the two groups (preserved and reduced TFF1 immunoexpression), the median survival times of animals with preserved TFF1 immunoexpression was 30 days and the median survival times of animals with reduced TFF1 immunoexpression was 12.0 days. Kaplan–Meier survival analysis revealed no significant survival differences between the two cohorts (log-rank test, *p* = 0.5976). 

## 4. Discussion

The role of TFF1 or pS2 in gastric carcinogenesis has been investigated in several human and mouse models, and it has been proposed that it might act as a tumor suppressor factor [24,28]. Previously, Campbell and Jabbes [29] have sequenced canine and feline TFFs cDNAs derived from gastric (TFF1, TFF2) and colonic (TFF3) mucosa RNA and showed that the majority of the deduced amino acid sequences of canine and feline TFFs obtained were in agreement with those of other mammalian species (e.g., human, rat, mouse, cow, pig, sheep), supporting the theory that the dog and cat may prove to be useful models for the study of trefoil peptides in various pathologies, such as IBD and gastrointestinal carcinomas. The present study was carried out to investigate whether there is a relationship between TFF1 immunoreactivity and canine gastric carcinogenesis.

Our study was conducted on eight GPs and thirty-one GCs. Reinforcing previous data, herein the dogs affected with GPs were males [1]. The most common was the hyperplastic type, and the lesions were preferentially located in the gastric antrum. Concerning GCs, the male predilection and mean age recorded in this series agree with those of others [1,35]. In literature, a breed predisposition has been reported in belgian shepherd dogs, rough collies, staffordshire terriers, chow-chows, and standard poodles [1]. In the present study, crossbreed dogs predominate (five dogs or 16.1%) followed by chow-chow (four dogs or 12.9%). In this series, there was a predominance of signet ring cell (10/31) and diffuse type (20/31) carcinomas, according with the human WHO and Lauren classifications, respectively. Our findings are keeping with previous studies in dogs [8,35,36]. Furthermore, our data support the preferential location of GCs in the antral region [8,35]. Additionally, the mean survival time for dogs with GCs was 31.2 days, which goes towards the reported 35 days proposed by Swann and Holt [37].

Our findings in normal canine gastric mucosa are in accordance with those previously reported regarding normal human [12,25,34] and mouse gastric tissues [38]. TFF1 was ubiquitously expressed in the superficial foveolar epithelium of the oxyntic and antral mucosa of the dogs. In addition, the predominantly cytoplasm subcellular location, with a more intense immunostaining in the apical region, supports the notion that TFF1 gene encodes a secretory protein. Similarly, to human studies [12,25,26,34], we also observed weak immunostaining for TFF1 in antral glands. Previously, Ren et al. [39] evaluated the molecular forms of TFF1 in human normal gastric mucosa and found three patterns of TFF1: monomer (6.5 kDa), dimmer (13 kDa), and TFF1 compound (about 21 kDa), suggesting that the biological activity of TFF1 may be related to formation of homologous dimmer or other oligomers composed of heterogenous proteins. In the present study, a Western blot analysis using an anti-estrogen inducible protein pS2 rabbit monoclonal antibody (clone EPR3972) demonstrated that in normal canine gastric mucosa, TFF1 has a molecular weight near 15 and 20 kDa. Collectively, our observations validate the specificity and cross-reactivity of this specific antibody with canine tissues. We speculate that TFF1 found in normal gastric mucosa is composed of TFF1 and its receptor, transport protein, or some glycoprotein. Further studies are needed to explore the molecular forms of TFF1 in normal canine gastric mucosa and abnormal gastric tissues. 

Machado et al. [12] and Nogueira et al. [34] evaluated the expression of TFF1 in human GPs and found a consistent expression of TFF1 in all the hyperplastic polyps analyzed (n = 10). In the present study, the expression of TFF1 in GPs was also consistently observed in all cases, displaying an immunopattern analogous to that of normal canine gastric mucosa. Our findings are in accordance with human studies and reinforce our previous investigations [10,16] in which canine GPs are considered merely proliferative lesions, at least phenotypically very similar to normal canine gastric epithelium. 

In the present study, we found two GCs with foci of IM and the one only available for TFF1 testing did not reveal any immunoexpression in the metaplastic cells. The expression of TFF1 in normal gastric mucosa together with the absence of immunoreactivity in IM fit the hypothesis that TFF1 can be used as a marker of gastric differentiation, as observed in human studies [25,26]. However, further studies are needed to prove the usefulness of TFF1 protein as a marker of gastric differentiation in dogs.

In GCs, TFF1 expression was found reduced in 74.2% of the cases, a frequency that is close to that reported by Moss et al. [40] (>70%) but above that reported by Wu et a., [41], Im et al. [42], and Muller and Borchard [26] (58.3%, 53.8%, and 48%, respectively) in human GCs series. The difference in the percentage obtained in this study and those from human GCs series may be related to the species in question (human vs. dog), to the antibodies against TFF1 used, and to the scoring system adopted for the immunoreactivity evaluation.

Tanaka and colleagues [43] found, in a series of 182 human GCs, that low expression of TFF1 was significantly correlated with deeper invasion of the tumor. They also found in the in vitro analysis that the invasive activity of gastric cancer cells increased significantly in TFF1-deficient cells compared with the control cells [43]. In the present study, the expression of TFF1 was observed to gradually decreased from mucosa to deeper layers, suggesting that the loss or reduction of this protein may confer an invasive phenotype to canine gastric neoplastic cells and thus promote cancer progression. Similar findings were discussed by Sunagawa et al. [44] who reported lost or decreased expression of TFF1 in the tumor invasion front of human pancreatic ductal adenocarcinomas (PDAC). Based on these findings, they speculated that the loss of TFF1 resulted in the epithelial–mesenchymal transition (EMT) of tumor cells and, therefore, upregulation of TFF1 might inhibit the EMT in cancer cells. During EMT, epithelial cells change their phenotype, exhibiting a reduction in cell–cell contacts, loss of polarity, increased cell motility and invasiveness, repression of epithelial cell markers (e.g., epithelial cells adhesion molecule (EpCAM), cytokeratin (CK), or E-cadherin), and aberrant upregulation of certain mesenchymal markers (e.g., vimentin and N-cadherin) [45]. Indeed, a recent study demonstrated that the overexpression of TFF1 inhibits EMT through regulation of TGF-β in gastric cancer cells. The authors found that elevated TFF1 levels induced the expression of E-cadherin, and reduced the expression of vimentin, N-cadherin, and others well-known repressors of E-cadherin expression. 

We observed a significant decrease in the TFF1 expression level from normal gastric mucosa to GPs and to GCs. These findings suggest that TFF1 may have a role in maintenance of normal canine gastric mucosal integrity, and that the loss of TFF1 may be associated with the malignant transformation of gastric mucosa. Hence, we speculated that TFF1 may act as a tumor suppressor during canine gastric carcinogenesis, as previously mentioned for human and mouse species [24]. In human gastric cancer, there are two probable causes for the low expression or absence of TFF1: 1) potential genetic alterations, including gene mutation, loss of heterozygosity (LOH), and DNA methylation; 2) poorly differentiated cells and glands, which are extremely altered to secrete TFF1 [39,43,46]. Future studies are needed in order to elucidate the precise mechanism behind reduced TFF1 expression and its involvement in canine gastric carcinogenesis.

Controversial data have been found regarding the expression of TFF1 in human GCs based on histological subtype. Im and coworkers [42] reported a much higher frequency of TFF1 expression in undifferentiated and diffuse type carcinomas compared with differentiated and intestinal type carcinomas. In contrast, Shi et al. [47] found that the expression level of TFF1 was lower in poorly differentiated carcinomas than in well-differentiated carcinomas. In the present study, the frequency of carcinomas displaying a reduced expression of TFF1 was higher in poorly/undifferentiated carcinomas (80%) and, in indeterminate and diffuse type (80% and 80%, respectively) carcinomas than in well-differentiated and intestinal type carcinomas (50%), respectively. However, this difference was not statistically significant. 

We found no significant difference between TFF1 expression and the presence of neoplastic emboli and metastatic disease. These findings concur with previous reports [42,48] in human GCs but contrast with those of Machado et al. [25] who found a significant association between TFF1 expression and lymph node metastases. However, we observed that the expression of TFF1 in neoplastic emboli was generally higher than that of the corresponding primary lesion. Given the higher expression of TFF1 in circulating neoplastic cells, we speculate that TFF1 can be detected in the serum, and, therefore, might be applicable in clinical practice as a non-invasive biomarker for the screening of gastric cancer progression. We also found that TFF1 expression in metastases was equal to or greater than that of the primary tumor. The expression of TFF1 in metastatic lesions suggests that neoplastic cells that have traveled to other tissues try, as soon as they arrive, to maintain or re-establish at least some of their original biological properties, namely by restoring common TFF1 expression of normal gastric epithelial cells. In the future, it would be interesting to explore this theory, analyzing a greater number of cases of patients with metastases.

In domestic animals, although some potential prognostic markers have been investigated in canine GC (such as HER-3, HER-2, EGFR, and KRAS), its value as prognostic indicators were not demonstrated [35,49] probably due to small numbers of reported neoplasms and also the lack of a complete clinical history and follow-up. In the present study we investigate whether the immunohistochemical evaluation of TFF1 carries any meaningful prognostic information for canine GCs. A small difference that did not reach statistical significance between the median survival times of dogs with preserved TFF1 (30 days) and reduced TFF1 tumors (12days) was found. The lack of correlation between TFF1 expression and survival of human gastric cancer patients was reported by several authors [25,26,41]; however, Suarez et al. [48] reported that high intratumoral TFF1 levels were significantly associated with an unfavorable outcome.

Some limitations of this study are the reduced number of cases, subjected to different sampling methods (partial vs. full-thickness biopsies), and submitted to different and not standardize clinical approaches. Additionally, the survival time may not be exact since the cases submitted to euthanasia were based on clinical decisions and may cover different stages of the oncological disease. Notwithstanding, this investigation represents an important contribution for the study of canine gastric carcinogenesis, as it gathers epidemiological and histopathological information from a group of dogs whose gastric lesions were the target of a pioneer study using an important antibody considered a tumor suppressor in cases of human gastric cancer.

## 5. Conclusions

In conclusion, the present study revealed a significant reduction in the expression of TFF1 in GCs when compared with GPs and normal gastric mucosa, which suggests that TFF1 may act as a tumor suppressor during canine gastric carcinogenesis. Additionally, there was a gradual decrease in TFF1 expression from mucosa to deeper layers, suggesting that the loss or reduction of this protein may confer an invasive phenotype on canine gastric neoplastic cells and thus promote cancer progression. Further studies on a larger number of cases and clinical follow-ups are necessary to define, with greater scientific accuracy, the clinical significance of TFF1 and its usefulness as a prognostic indicator.

The pathological and behavioral similarities between spontaneous canine and humans’ carcinomas make it logical to assume that dogs may be a useful model for human gastric cancer.

## Figures and Tables

**Table 1:** Animal epidemiological data (breed, age, sex) and characteristics of the specimens included in the present study.

Case	Breed	Sex/Age (Years)	Location of the Lesion	Histological Diagnosis	
1	Boxer	M/10	N/A	Normal mucosa	
2	Basset hound	F/9	N/A	Normal mucosa	
3	Yorkshire terrier	M/3	N/A	Normal mucosa	
4	Crossbreed	M/16	Body	Inflammatory polyp	
5	Poodle	M/12	Antrum	Hyperplastic polyp	
6	Boxer	M/10	Antrum	Hyperplastic polyp	
7	German shepherd	M/9	Antrum	Inflammatory polyp	
8	Irish setter	F/12	Antrum	Hyperplastic polyp	
9	Argentine mastiff	F/9	Antrum	Hyperplastic polyp	
10	Poodle	M/13	Antrum	Inflammatory polyp	
11	Crossbreed	F/16	Antrum	Hyperplastic polyp	
				WHO [31]	Lauren [32]
12	Basset hound	F/12	Antrum	Tubular	Intestinal
13	Cocker spaniel	M/13	Antrum	Signet ring cell	Diffuse
14	Chow-chow	F/11	Antrum	Mixed	Indeterminate
15	Shih Tzu	F/10	Antrum	Poorly cohesive	Diffuse
16	Chow-chow	M/10	Antrum	Signet ring cell	Diffuse
17	English bulldog	M/6	Body	Signet ring cell	Diffuse
18	Labrador retriever	F/14	Body	Tubular	Intestinal
19	Shar-pei	M/5	Body	Signet ring cell	Diffuse
20	Belgian shepherd	F/11	Body	Mixed	Indeterminate
21	Golden retriever	M/14	Body	Signet ring cell	Diffuse
22	Labrador retriever	M/8	Antrum	Mixed	Indeterminate
23	Siberian husky	F/12	Antrum	Tubular	Intestinal
24	Crossbreed	M/10	Body	Mucinous	Diffuse
25	Crossbreed	F/8	Body	Poorly cohesive	Diffuse
26	Siberian husky	M/13	Antrum	Tubular	Intestinal
27	Crossbreed (X German shepherd)	F/13	Body	Poorly cohesive	Diffuse
28	Akita	M/9	Body	Poorly cohesive	Diffuse
29	Collie	M/11	Body	Mixed	Indeterminate
30	Alaska malamute	M/6	ID	Signet ring cell	Diffuse
31	Golden retriever	M/10	Antrum	Signet ring cell	Diffuse
32	Chow-chow	M/9	Antrum	Poorly cohesive	Diffuse
33	Pointer	M/11	Body	Signet ring cell	Diffuse
34	Collie	M/11	Body and antrum	Poorly cohesive	Diffuse
35	Boxer	M/7	Antrum	Signet ring cell	Diffuse
36	Crossbreed	F/7	Antrum	Poorly cohesive	Diffuse
37	Chow-chow	M/6	Body	Mucinous	Diffuse
38	West highland white terrier	F/13	Antrum	Signet ring cell	Diffuse
39	Standard Poodle	M/8	Antrum	Mixed	Indeterminate
40	Crossbreed (X Poodle)	F/9	Antrum	Papillary	Intestinal
41	Miniature Poodle	F/14	Antrum	Tubular	Intestinal
42	German shepherd	M/12	Body and antrum	Poorly cohesive	Diffuse

M-male, F–female; N/A—not applicable; ID—data was not available.

**Table 2:** Association between TFF1 expression and clinicopathological features in 31 canine gastric carcinomas.

Parameter	No. of Cases	TFF1 Immunoreactivity		
		Preserved N = 8 (25.8%)	Reduced N = 23 (74.2%)	*p*-Value
Sex				
Male	**19**	4 (21.1)	15 (78.9)	0.447
Female	**12**	4 (33.3)	8 (66.7)	
**Age, years**				
<10	**12**	2 (16.7)	10 (83.3)	0.355
≥10	**19**	6 (31.6)	13 (68.4)	
**Tumor location ^1^**				
Antrum	**16**	6 (37.5)	10 (62.5)	0.317
Body	**12**	2 (16.7)	10 (83.3)	
Body + antrum	**2**	0 (0)	2 (100)	
**Histological diagnosis**				
*WHO* classification [31]				
Well-differentiated	**6**	3 (50)	3 (50)	0.132
Poorly/undifferentiated	**25**	5 (20)	20 (80)	
Lauren [32]				
Intestinal	**6**	3 (50)	3 (50)	0.229
Diffuse	**20**	4 (20)	16 (80)	
Indeterminate	**5**	1 (20)	4 (80)	
**Depth of tumor invasion ^2^**				
Muscular	**10**	3 (30)	7 (70)	0.457
Serosa	**12**	2 (16.7)	10 (83.3)	
**Neoplastic emboli**				
Present	**18**	3 (16.7)	15 (83.3)	0.171
Absent	**13**	5 (38.5)	8 (61.5)	
**Metastatic lesions ^3^**				
Present	**9**	1 (11.1)	8 (88.9)	0.186
Absent	**17**	6 (35.3)	11 (64.7)	

^1^ Information regarding tumor location was impossible to obtain in one case. ^2^ For statistical analysis only full-thickness biopsies were included. ^3^ Information regarding metastatic lesion was impossible to obtain in five cases.

## Data Availability

The data presented in this study is contained within the manuscript.

## Supplementary Materials

The following are available online at https://www.mdpi.com/article/10.3390/ani11102855/s1, Figure S1: Western blot analysis of anti-estrogen inducible protein pS2 rabbit monoclonal antibody in normal canine gastric mucosa. (1) Normal canine gastric mucosa protein extract. Presence of a dominant band near 15 and 20 kDa.
