# Supplementary material for: Immunoexpression of Trefoil Factor 1 in Non-Neoplastic and Neoplastic Canine Gastric Tissues
Source: Animals (Basel). 2021 Sep 29;11(10):2855. doi: 10.3390/ani11102855 (PMC8532865; doi:10.3390/ani11102855)
Supplement: Supplementary file 1 [file animals-11-02855-s001.zip › animals-1365633 - supplementary materials.pdf]

animals

MDPI

Supplementary Material

Immunoexpression of Trefoil Factor 1 in Non-Neoplastic and Neoplastic Canine Gastric Tissues

Ana R. Flores [1,2,3], Marisa Castro [1], Alexandra Rêma [1], João R. Mesquita [4], Marian Taulescu [5,6,*], Fátima Gärtner [1,2,7], Fernanda Seixas [3] and Irina Amorim [1,2,7]

1 Department of Pathology and Molecular Immunology of the Institute of Biomedical Sciences Abel Salazar (ICBAS), University of Porto; Rua Jorge Viterbo Ferreira nr. 228, 4050-313 Porto, Portugal; anaruteflores@gmail.com (A.R.F.); mmcastro@icbas.up.pt (M.C.); alexandra.rema@gmail.com (A.R.); fgartner@ipatimup.pt (F.G.); iamorim@ipatimup.pt (I.A.)
2 Institute of Pathology and Molecular Immunology of the University of Porto (IPATIMUP); Rua Dr Roberto Frias s/n, 4200-465 Porto, Portugal
3 Animal and Veterinary Research Center (CECAV), Associate laboratory AL4AnimalS, University of Trás-os-Montes e Alto Douro (UTAD), Quinta dos Prados, apartado 1013, 5001-801 Vila Real, Portugal; fseixas@utad.pt
4 Epidemiology Research Unit (EPIUnit), Instituto de Saúde Pública da Universidade do Porto (ISPUP); Rua das Taipas, n.º 135, 4050-600 Porto, Portugal; jmesquita@outlook.com
5 Department of Pathology, Faculty of Veterinary Medicine, University of Agricultural Sciences and Veterinary Medicine, 400372 Cluj-Napoca, Romania
6 Synevovet Laboratory, 81 Pache Protopopescu, 021408 Bucharest, Romania
7 i3S-Instituto de Investigação e Inovação em Saúde, Universidade do Porto; Rua Alfredo Allen, 4200-135 Porto, Portugal
* Correspondence: marian.taulescu@usamvcluj.ro

Citation: Flores, A.R.; Castro, M.; Rêma, A.; Mesquita, J.R.; Taulescu, M.; Gärtner, F.; Seixas, F.; Amorim, I. Immunoexpression of Trefoil-Factor 1 in Non-Neoplastic and Neoplastic Canine Gastric Tissues. *Animals* **2021**, *11*, 2855. https://doi.org/10.3390/10.3390/ani11102855

Academic Editor: Emir Hadzijusufovic

Received: 18 August 2021
Accepted: 26 September 2021
Published: 29 September 2021

Publisher's Note: MDPI stays neutral with regard to jurisdictional claims in published maps and institutional affiliations.

Copyright: © 2021 by the authors. Licensee MDPI, Basel, Switzerland. This article is an open access article distributed under the terms and conditions of the Creative Commons Attribution (CC BY) license (http://creativecommons.org/licenses/by/4.0/).

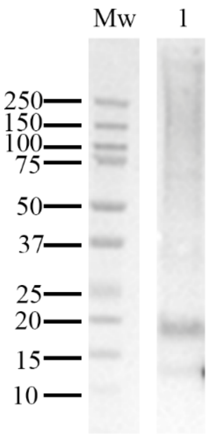

Figure S1. Western blot analysis of Anti-estrogen inducible protein pS2 rabbit monoclonal antibody in normal canine gastric mucosa. (1) Normal canine gastric mucosa protein extract. Presence of a dominant band near 15 and 20 kDa.
